# Scalable funding of Bitcoin micropayment channel networks

**DOI:** 10.1098/rsos.180089

**Published:** 2018-08-29

**Authors:** Conrad Burchert, Christian Decker, Roger Wattenhofer

**Affiliations:** 1 ETH Zurich, Zurich, Switzerland; 2 Blockstream Inc., San Francisco, CA, USA

**Keywords:** Bitcoin, lightning network, micropayment channel networks

## Abstract

The Bitcoin network has scalability problems. To increase its transaction rate and speed, micropayment channel networks have been proposed; however, these require to lock funds into specific channels. Moreover, the available space in the blockchain does not allow scaling to a worldwide payment system. We propose a new layer that sits in between the blockchain and the payment channels. The new layer addresses the scalability problem by enabling trustless off-blockchain channel funding. It consists of shared accounts of groups of nodes that flexibly create one-to-one channels for the payment network. The new system allows rapid changes of the allocation of funds to channels and reduces the cost of opening new channels. Instead of one blockchain transaction per channel, each user only needs one transaction to enter a group of nodes—within the group the user can create arbitrarily many channels. For a group of 20 users with 100 intra-group channels, the cost of the blockchain transactions is reduced by 90% compared to 100 regular micropayment channels opened on the blockchain. This can be increased further to 96% if Bitcoin introduces Schnorr signatures with signature aggregation.

## 1. Introduction

The increasing popularity of Bitcoin and other blockchain-based payment systems leads to new challenges, in particular, regarding scalability and transaction speed. During peaks of incoming transactions, the blockchain cannot process them fast enough and a backlog is created. A second major problem is transaction speed, the time from initiating a transaction until one can assume that the transaction has concluded, and is thus irreversible. With inter-block times typically in the range of minutes and multiple blocks needed to reasonably prevent double spending, transactions take minutes to hours until the payment is confirmed. This may be acceptable for long-term Bitcoin investors, but not for everyday shopping or interacting with a vending machine [1].

To solve both scalability and speed, micropayment channel networks have been proposed [2,3]. A micropayment channel provides a way to trustlessly track money transfers between two entities off-blockchain with smart contracts. If both parties are honest, they can commit the total balance of many transfers in a single transaction to the blockchain and ignore the smart contracts. If a node crashes or stops cooperating otherwise, the smart contracts can be included in the blockchain and enforce the last agreed-on state.

If two parties do not have a channel, a network of multiple micropayment channels can be used together with a routing algorithm to send funds between any two parties in the network. Hashed timelocked contracts (HTLCs) provide a scheme to allow atomic transfers over a chain of multiple channels [2–4].

As micropayment channel networks will keep most transactions off the blockchain, blockchain-based currencies may scale to magnitudes larger user and transaction volumes. Also, micropayment channel networks allow for fast transactions, as a transaction happens as soon as a smart contract is signed—the blockchain latency does not matter.

### 1.1. Challenges

Micropayment channel networks create new problems, which have not been solved in the original publications [2,3]. We identify two main challenges—the blockchain capacity and locked-in funds.

Even with increases in block size, it was estimated that the blockchain capacity could only support about 800 million users with micropayment channels due to the number of on-chain transactions required to open and close channels [5]. A large-scale adoption of micropayment channel networks, where e.g. Internet of Things devices have their own Bitcoin wallet, brings the blockchain to its limit.

Two parties cooperating in a channel must lock funds into a shared account. The locked-in funds should be sufficient to provide enough capacity for peaks of transactions. There is a conflict of the two aims to have a low amount of funds locked up in a channel, while at the same time being flexible for these peaks.

We will present a solution that improves on both problems. Payment channels will not appear in the blockchain, except in the case of disputes. Users will be able to enter the system with one blockchain transaction and then open many channels without further blockchain contact. Funds are committed to a group of other users instead of a single partner and can be moved between channels with just a few messages inside this collaborating group, which reduces the risk, as an unprofitable connection can be quickly dissolved to form a better connection with another partner. By hiding the channels from the blockchain, a reduction in blockchain space usage and thus the cost of channels is achieved. For a group of 20 nodes with 100 channels in between them, this can save up to 96% of the blockchain space.

The channels created inside these groups work in the same way as regular micropayment channels; therefore, members of such a group can forward payments over a larger payment network of regular channels, founded either directly on the blockchain or within other groups. This property enables easy deployment in an existing payment network.

## 2. Ingredients

For completeness, this section describes the previous work we are building on.

### 2.1. Blockchain transactions

The concept of a blockchain to store transactions in a decentralized payment system was introduced by Satoshi Nakamoto in 2008 [6]. The blockchain is a distributed append-only ordered list of transactions. To append a transaction to the blockchain, it is broadcast into the network of miners. We will use broadcast as a synonym for appending a transaction to the blockchain; we are waiting for enough confirmations to ensure that a blockchain transaction is irreversible with high probability.

Each transaction consists of inputs and outputs. An output is an amount of currency and a spending condition, e.g. specified in the Bitcoin Script language. An input is a reference to an existing, unspent output of another transaction and a proof fulfilling the spending conditions of the referenced output.

A useful option of this design is to create an output containing *n* public keys, which can be spent with signatures of *m* of the corresponding private keys, known as an m-of-n OP_CHECKMULTISIG or just multisignature output. This implements a shared account of *n* entities, which can be spent with the support of *m* of those entities.

### 2.2. Micropayment channels

**Figure 1.**
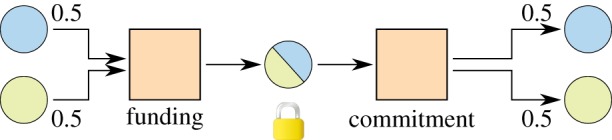
Construction of a micropayment channel. The boxes are transactions or a number of transactions and the circles are outputs. The colours in the circles describe whose signatures are needed to spend those outputs. To spend an output belonging to multiple parties, all of the parties must sign. The lock indicates unspent transaction outputs on the blockchain while the channel is open.

A micropayment channel is a set-up where two parties have created the means to send each other currency without contacting the blockchain. The construction principle is shown in figure 1.

**Figure 2.**
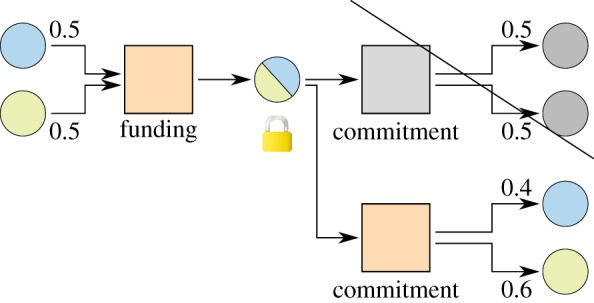
Update of a micropayment channel. A new commitment transaction replaces the old one. As long as it is ensured, the old commitment cannot be broadcast, 0.1 BTC have now changed ownership from blue to green.

The commitment is signed before the funding transaction to ensure that no funds can be taken hostage by one party, as the other party already holds the means to recover its stake. Both parties can close the channel at any time by broadcasting the prepared commitment. As the opposing party cannot spend from the shared account without both signatures, the funds are safe and the broadcast of the commitment can be delayed to a later point in time. Given a scheme to replace transactions, the channel can now be used to transfer funds by replacing the commitment transaction with new commitment transactions, which change the amount of currency sent to each party, as shown in figure 2. It is important to ensure that the old version of the commitment transaction cannot be used any more. We will look at methods to accomplish this in the next subsections.

The amount of locked funds determines the maximum imbalance between sent and received funds, until all funds are with a single partner only. This is the capacity of the channel. When a channel's capacity is depleted, currency must move in the other direction or the channel needs to be closed and reopened on the blockchain with additional funds.

### 2.3. Transaction replacement using timelocks

Channels which replace transactions using timelocks are known as duplex micropayment channels [2].

**Figure 3.**
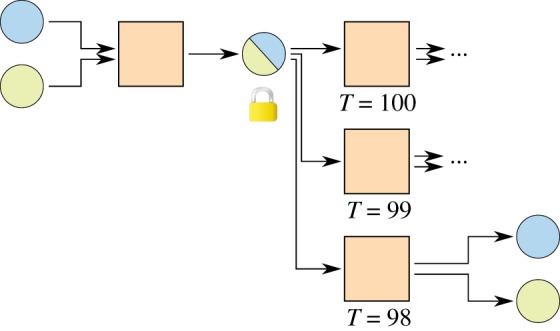
Micropayment channel with timelocks. The commitment with the lowest timelock can be included in the blockchain before the others.

Figure 3 shows a simple micropayment channel with timelocks. The first commitment transaction is created with a timelock of 100 days, meaning it cannot be appended to the blockchain until 100 days have passed. The second commitment transaction is created with a timelock of 99 days and spends the same funds, so it will be valid first and if anyone spends it during the first day, the outdated commitment transaction will never have a time where it can be broadcast, as the referenced output will have been spent already. Subsequent commitment transactions use lower timelocks, always having only one transaction which can be broadcast first.

**Figure 4.**
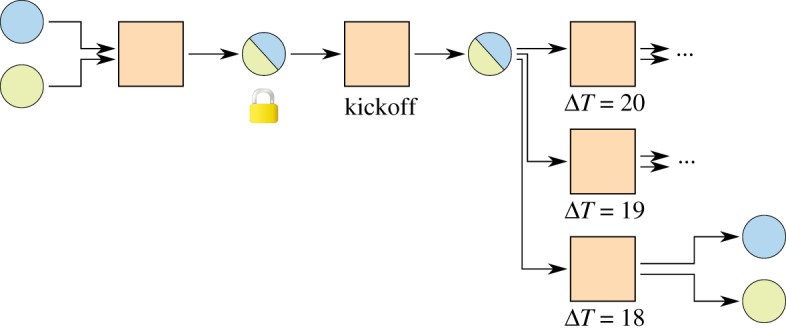
Micropayment channel with relative timelocks. Timelocks count relative to the inclusion of the previous transaction into the blockchain. No counters start until the kickoff transaction has been broadcast.

A channel constructed this way has to be closed by broadcasting the newest commitment transaction as soon as the first timelock has elapsed, limiting the maximum lifetime of a channel. With relative timelocks [7,8], this problem can be solved elegantly. Figure 4 introduces a kickoff^1^ transaction. Timelocks only start ticking as soon as the kickoff transaction is broadcast, resulting in a potentially unlimited lifetime of a channel.

**Figure 5.**
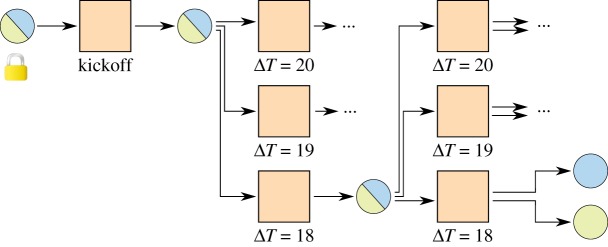
Invalidation tree with relative timelocks. The lowest path is the currently active one. The rest of the tree can be pruned, as it will never be valid.

Still, one quickly runs out of time by doing transactions in the channel, each requiring a smaller timelock on the commitment transaction. This was solved with a tree of transactions [2] as shown in figure 5.^2^ At any point in time only the path where all transactions have the lowest timelock of their siblings can be broadcast. In this way, many commitment transactions can be created before the timelocks get too low and the channel cannot be updated any more.

Implementations of the transactions according to figure 5 can be found in appendix A.

### 2.4. Transaction replacement using punishments

A variant of micropayment channels, known as lightning channels, uses revocable transactions to replace the commitments [3,9]. Each commitment consists of two transactions, one per user in the channel. A party can give up its personal transaction by revealing a secret, which allows the opponent to punish it in the case that it broadcasts the transaction afterwards.

## 3. Channel factories

As our main contribution, we introduce a new layer between the blockchain and the payment network, giving a three-layered system. In the first layer, the blockchain, funds are locked into a shared ownership between a group of nodes. The new second layer consists of multi-party micropayment channels we call channel factories, which can quickly fund regular two-party channels. The resulting network provides the third layer, where regular transfers of currency are executed.

Similar to regular micropayment channels, multi-party channels can be implemented with either timelocks or punishments for dishonest parties. Our implementation with timelocks scales much better to larger participant numbers, hence we will focus on it. The regular micropayment channels of the third layer can be punishment based or timelock based independent from the implementation of the multi-party channels of the second layer.

**Figure 6.**
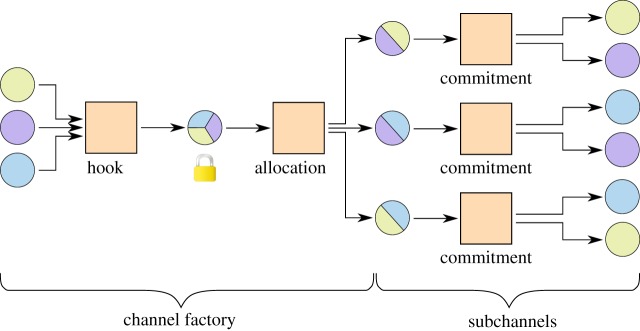
A three-party channel factory for three subchannels. The allocation and the commitments are replaceable transactions. The subchannels can be updated by the two collaborating parties by creating new commitments in a subchannel. All three parties together can collaborate to replace the allocation and thus create new and different two-party micropayment channels without contact to the blockchain.

Figure 6 shows an example channel factory of three parties that funds pairwise one-to-one channels.

We formally define some concepts.

**Definition 3.1 (Funding transaction).**

A funding transaction is a blockchain transaction with an OP_CHECKMULTISIG output that is used to lock funds into a shared ownership between the *p* collaborating parties.

Note that there are two types of funding transactions in the new system, funding a multi-party channel and funding the layer three two-party channels.

**Definition 3.2 (Hook transaction).**

The hook transaction is the funding transaction of the multi-party channel. It locks the funds of many parties into a shared ownership.

**Definition 3.3 (Allocation).**

The allocation is one transaction or a number of sequential transactions that take the locked funds from a multi-party channel as an input and fund many multi-party channels with their outputs.

The allocation effectively replaces the funding transactions of a number of two-party channels.

**Definition 3.4 (Commitment).**

A commitment is a transaction or a number of transactions that return the funds of a two-party channel to their owner.

Commitments are already known from two-party channels.

The channel is constructed by first creating all transactions of the initial state, then signing all except the hook and finally signing and broadcasting the hook. Signing the hook last ensures that the funds can be returned to their owners the in case that one party stops cooperating. After the hook is included in the blockchain and enough confirming blocks have been received, the channel can be used.

To implement the described set-up, the known constructions of payment channels can be extended. The hook transaction is a simple blockchain transaction which takes inputs from all users and creates one n-of-n OP_CHECKMULTISIG output, which can be spent with the signatures of all parties. The commitments include just two parties, thus the known implementations with timelocks or revocable transactions from §2 can be used directly. However, we need a new scheme for the allocations, as they need to be replaced in a trust-free way as well, but include more than two parties.

### 3.1. Replaceable allocations

**Figure 7.**
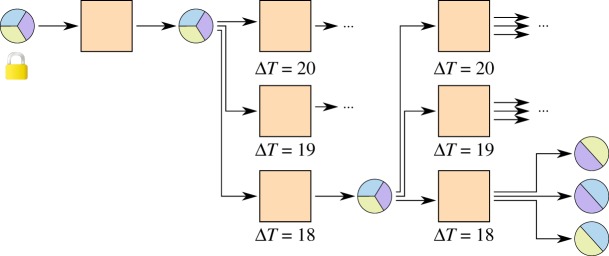
Allocation of a three-party channel factory. The invalidation tree can have any depth or degree of nodes. Timelocks start ticking as soon as the previous transaction is included in the blockchain. Each transaction can be broadcast after the relative locktime has elapsed.

Replaceable transactions with many parties can be implemented similarly to two-party channel commitments based on timelocks with an invalidation tree and a kickoff transaction at the root, which starts the timers when broadcast to the blockchain. The leaves of the invalidation tree create the two-party shared accounts. The principle is shown in figure 7.

Note that the order of the replacement of transactions is important. One should always have a state where the path of lowest timelocks does not end in unsigned transactions. When a new path is created in the tree, the first transaction which diverges from the old active path must be signed last, so the rest of the path is already valid and the whole new path replaces the old path atomically.

It is easy to show that there is no risk to the involved parties. Assuming that at least one party tries to broadcast transactions, when the timelocks have elapsed, only one path of the tree will ever be broadcastable, apart from situations where a channel update is in progress. While a new path is being created, there is a brief period where some parties already have the new path fully signed, while the other parties are missing signatures. This is not a problem, as this state is temporary and cannot be abused, as long as the receiver of a transaction does not regard a transfer as complete before he has received all new signatures.

Most of the tree can be pruned, thus the memory footprint is small. While a reallocation is in progress, new commitments can be made to the subchannels. To ensure that they are valid indifferent of whether the new allocation succeeds, commitments should be made on both the old and new subchannels. The details of the protocol to update an allocation will be discussed later in §3.6.

Bitcoin Script implementations of the transactions are found in appendix A.

### 3.2. Settlement

**Figure 8.**
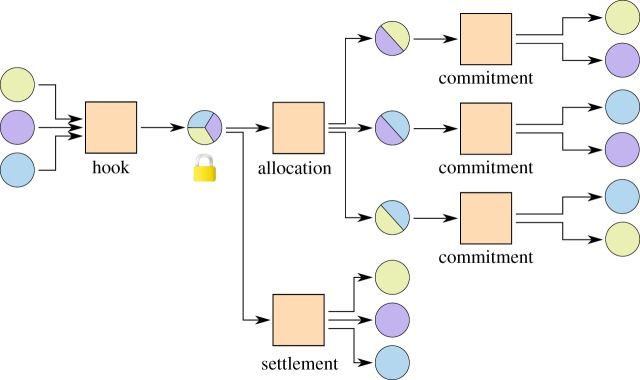
Settlement of a channel factory. Subchannels only appear on the blockchain in the case of conflicts.

When the involved parties cooperatively decide to close a channel factory, they can create and broadcast a settlement transaction, which pays out the current stake of each party directly from the shared account without a timelock, replacing the allocation and removing the locked funds (figure 8). This way only two transactions appear on the blockchain, the hook and the settlement, which saves blockchain space and hides the unnecessary information from the public. The protocol to create a settlement is simple. If one node decides to close the channel factory, it broadcasts this decision to all other nodes. Everyone stops updating the subchannels and broadcasts the sum of his current stake. This is enough information for each node to create and sign the settlement transaction and broadcast the signature. Nodes cannot profit from lying about their total stake, as if any node gave a number too high the total sum would exceed the locked-in funds of the factory and the settlement transaction would be invalid.

### 3.3. Moving funds

**Figure 9.**
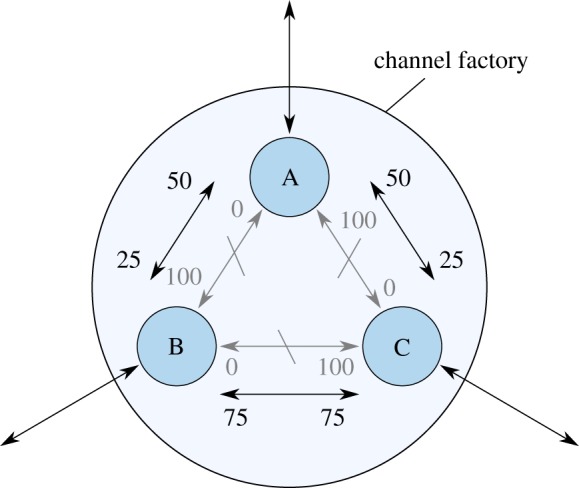
Rebalancing of three channels created in a channel factory. The depleted grey channels are replaced with new balanced ones by replacing the allocation transaction. Each party owns the same total stake as before. Furthermore, the nodes have moved more funds to the lower edge, e.g. because more traffic was measured there.

A channel factory can be used to rebalance channels which have become one sided. A new allocation is set up which replaces every channel with a balanced new one while keeping the total stake of each party the same. As an advantage, funds can also be moved between channels, new channels can be created or old ones removed, changing the network connectivity without contacting the blockchain. Figure 9 shows such a rebalancing where funds are simultaneously moved to a different channel.

### 3.4. Including a cold wallet in a channel

**Figure 10.**
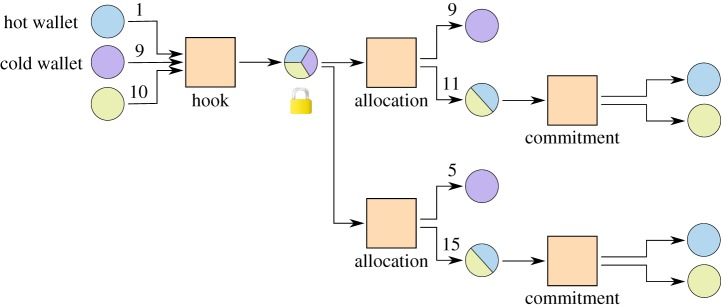
Connecting a cold wallet to a payment channel with a channel factory. By replacing the allocation transaction, the owner of the cold wallet can move money into the payment channel without blockchain interaction.

Another use case of a channel factory is to include a cold wallet^3^ in the creation process of a channel to later stock up the channel as shown in figure 10. The construction allows the cold wallet to be offline most of the time. When the owner wants to move money from or into the channel, the cold wallet can be brought online to create a new allocation. Updates to the subchannel do not need the cold wallet and are used for normal value transfers.

### 3.5. Leaving a group

**Figure 11.**
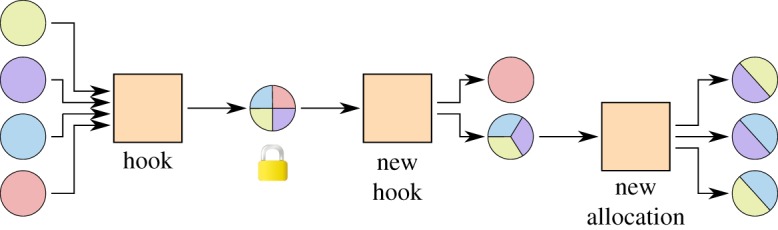
A channel after one node has left. The allocation was replaced with a new hook. After broadcasting the new hook, the leaving node can spend its money on the blockchain and the others can continue with the channel.

In large groups, there may be situations where some node wants to leave; however, the others would like to continue the channel. Instead of closing the channel factory and opening a new one, both actions can be combined into one transaction, as shown in figure 11.

### 3.6. Coordination of allocation updates

When a new allocation is created, the members of a channel factory need to coordinate the creation of a new allocation transaction and all transactions to make the new subchannels of this new allocation. Owing to the number of involved parties, this might take a considerable amount of time. However, this is not a problem, as normal channel operation can be continued as long as care is taken to make changes to the subchannels of both the old and the new allocation. An allocation update can be executed in the following order:

(i) A member decides that an update of the allocation is necessary, e.g. because it wants to move funds to another channel, and broadcasts to all nodes of the group that a new allocation should be created.
(ii) As soon as someone receives the allocation update request, he will issue a request to all his subchannel partners to use the current channel state as the base for the new allocation.
(iii) In each subchannel, the two cooperating parties decide on a starting state for the new subchannel and broadcast it to the group. Nodes can apply changes that move funds to other channels in this step.
(iv) Each node creates the new allocation transaction. These should all be identical, as they fund the same two-party shared accounts.
(v) The two cooperating parties of each subchannel create the subchannel commitment transactions and sign them. From this point on, they keep both subchannels based on the old and new allocation updated.
(vi) All nodes sign the new allocation and exchange signatures.
(vii) After receiving all signatures on the new allocation, a node can stop updating the subchannels based on the old allocation, as those cannot be executed any more.

From the view of any node, there are three states during this process. In the first state, the node knows that only the old allocation may come into effect. In the second state, the node has given away its signature on the new allocation, however, not received all signatures from the other nodes, thus it is uncertain which allocation might be executed on the blockchain. After receiving all signatures, the node can enforce the new allocation due to its lower timelock. By starting to apply changes to both the old and new subchannels before giving away its own signature on the new allocation, it is always ensured that the newest subchannel state is enforceable on the blockchain.

Note that it is clear that movements of funds are consistent, i.e. no node can create money by telling different partners different information about moving funds between channels, as the total sum must not exceed the locked funds of the group. A net gain for some party must result in a net loss for another party, which will refuse to sign the new allocation. Furthermore, if there are different versions of the new allocation, the signatures of some parties will not be applicable to the transactions of others and the new allocation cannot come into effect, as no one has a complete set of signatures. If this happens by error, the case can be resolved either by retrying with another new allocation or by giving up and eventually resolving the situation on the blockchain with an old allocation.

The described procedure uses broadcasts of subchannel sizes and signatures. This results in a communication overhead of *O*(*p*^2^), where *p* is the number of members in the channel factory. If this is considered too large, a leader can be chosen, e.g. the node with the smallest input index in the funding transaction of the channel factory. The leader can collect and distribute the information, reducing the number of messages to *O*(*p*). The time used by the protocol is constant.

### 3.7. Higher order systems

**Figure 12.**
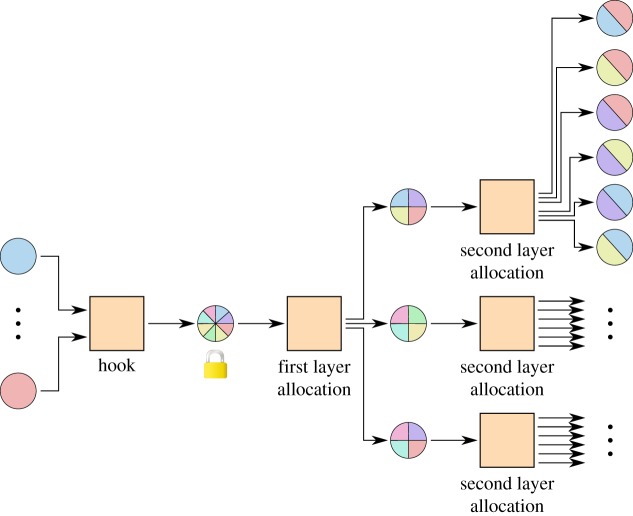
A multi-party channel of eight parties, which are divided into three overlapping subgroups of four parties each. Only signatures from four parties are needed to move money between channels inside one of the subgroups, but all eight nodes can be connected at least indirectly.

With larger groups, the coordination work required to sign a new allocation rises, but it is advantageous to create large groups to save blockchain space and have more partners for subchannels. It is possible to extend the system to more layers, each layer having less parties per shared account, as shown in figure 12.

This set-up uses the same number of signatures as a system with two independent groups, one to enter and one to leave per entity. However, with two independent groups, no channels between members of different groups would be possible without additional blockchain transactions. With higher order systems, multiple groups can be combined into one larger group, which can create overlapping subgroups. This allows us to create channels which enable paths between any two members of the larger group.

### 3.8. Risks

With a rising number of parties in a channel factory, the number of parties that can stop cooperating and close the channel rises, as anyone involved in the multi-party channel can broadcast the allocation to the blockchain. Afterwards the subchannels can still be used, as the funds are now locked in the two-party accounts, but the option to move funds between channels is lost. There is no personal advantage in unilaterally closing a channel, as the only difference is that higher mining fees are paid for the increased blockchain space, thus everyone loses. A selfish user should always prefer a settlement solution in comparison to broadcasting the current path of the invalidation tree.

### 3.9. Signature aggregation

It has been proposed to introduce Schnorr signatures [10] in Bitcoin, which would enable signature aggregation.^4^ Signature aggregation allows us to combine many public keys into a single public key and many signatures into a single signature. N-of-n multisignature outputs could be created with just one public key and the corresponding input would contain a single signature, saving blockchain space. Furthermore, the transaction format could be modified to use a single signature, which signs the combination of the public keys of all inputs [11]. Channel factories use transactions with many public keys and would thus profit highly from these possibilities.

### 3.10. Fees

Higher order systems enable larger groups, where creating a new allocation in an upper layer might require a significant number of collaborating nodes. Nodes which would like to change the affiliation with subgroups could pay fees to everyone else in the group to incentivize help to update the allocation. As all subchannels are replaced, this is easily accomplished by creating larger channels everywhere the initiating party is not involved and reducing the initiating party's stake in its own channels. Integrated into the new channel state, this payment is atomically executed with the allocation update.

### 3.11. Combining channel factories

**Figure 13.**
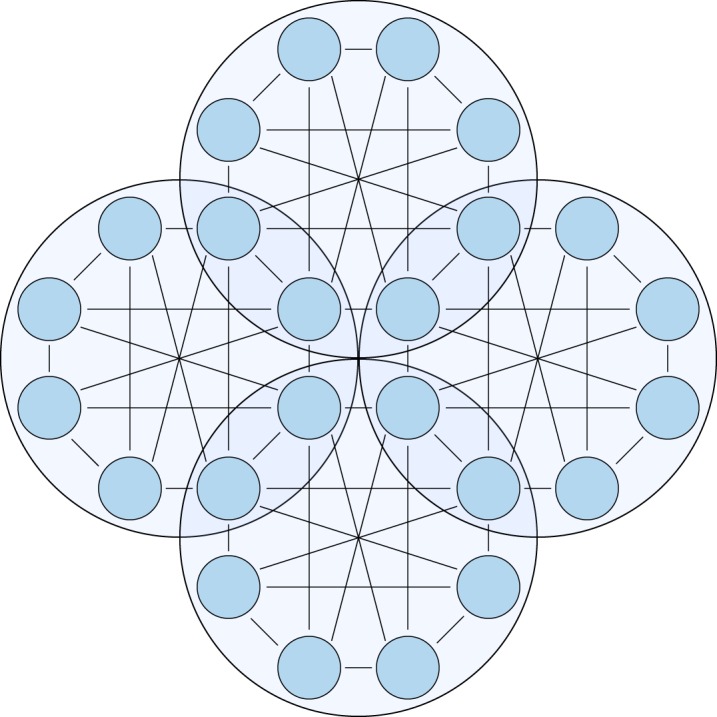
A possible structure of a payment network with four channel factories, each including eight members. The channel factories share a few users to allow the routing of payments over the borders of the groups. There exist four disjoint paths between any two nodes.

As it is unlikely that all users of the payment network will share a single channel factory, there will be some kind of connecting structure between the different groups in the network. As it is not possible to join an existing channel factory without additional cost, it is likely that new users joining the system will group together to create new channel factories. To connect to the rest of the network, they need to include a few nodes already connected in other channel factories. The result might look similar to figure 13. Members of multiple groups have a higher initial cost, as they need to be part of multiple hook transactions on the blockchain; however, they will also experience more traffic and can try to make profits by routing payments. Coordinating the creation of new groups and being such a connecting node might become a business model.

## 4. Evaluation

To evaluate the cost reduction, we assume that the largest part of the cost of a money transfer in the payment network results from the space occupied in the blockchain to create the channels. The price of blockchain space is regulated by the fee market and is paid per byte of transaction data, thus more complex transactions are more expensive. We will approximate how many bytes of blockchain space are used to create a single payment channel. As someone closing a channel unilaterally loses money, it can be assumed that few disputes will reach the blockchain. Hence, we only consider cooperatively closed channels.

Public keys and signatures comprise a large part of the transaction data. We use this to avoid lengthy byte counting of the Bitcoin transaction structures and define a simplified metric:

**Definition 4.1 (Blockchain cost).**

Assume all payment channels are closed in cooperation of the involved parties. The blockchain cost BC is the sum of the size of the public keys and signatures of the broadcast transactions during the lifetime of a channel.

We start by evaluating the system with the currently used ECDSA signatures and therefore without signature aggregation. On average, an ECDSA signature constitutes 72 bytes, a public key 33 bytes. Channel factories closed cooperatively only broadcast two transactions, the hook and the settlement. Each of the two transactions contains one signature and one public key per participant. Let *p* be the number of parties in the channel factory and *n* be the number of subchannels. The blockchain cost per subchannel is as follows:

$$\text{BC}(p,n)=\frac{33\times 2\times p+72\times 2\times p}{n}=210\times \frac{\;p}{n}.$$

To set this into context, we also calculate the blockchain cost in a system, where all one-to-one payment channels are opened directly on the blockchain. Both the funding and settlement of every channel each require two public keys and two signatures.

$${\text{BC}}_{\mathrm{simple}}=33\times 2\times 2+72\times 2\times 2=420.$$

If *p* = 3 entities form a second layer group to create *n* = 3 pairwise channels, their blockchain cost is 210, so they already save 50% of the blockchain space. With *p* = 20 parties and *n* = 100 subchannels, the blockchain cost of each channel is 42, which is 10% of the original cost.

With Schnorr signatures, only one signature is necessary to sign all inputs of the hook transaction, and one combined public key can be used for the output. The settlement can also use a single signature, but needs to provide the public key for each output. If Schnorr signatures are implemented with the ed25519 curve [12], which provides a similar security level as the current ECDSA implementation, a public key uses 32 bytes and a signature 64 bytes.^5^ This results in the following:

$${\text{BC}}_{\mathrm{Schnorr}}(p,n)=\frac{32\times (p+1)+64\times 2}{n}=\frac{32\times p+160}{n}.$$

One-to-one channels without a channel factory use one signature on the funding transaction, one public key on the hook, one signature on the settlement and two public keys on the settlement. This results in the following:

$${\text{BC}}_{\mathrm{simple},\mathrm{Schnorr}}=32\times 3+64\times 2=224.$$

With *p* = 3 parties in a channel factory with *n* = 3 subchannels, we calculate a blockchain cost of 85.3, an improvement of 62% compared to blockchain-funded channels. With *p* = 20 parties and *n* = 100 channels, the cost is 8, an improvement of 96%. It is clear that channel factories increase their usefulness with Schnorr signatures.

## 5. Related work

The need for scalability is well understood. Apart from simply changing the parameters [13,14], the efficiency of the original Bitcoin protocol still offers space for improvement [15–19].

Increasing the transaction speed without payment networks has been investigated. It was shown that double spending is easily achievable without doing any mining if the receiver is not waiting for any confirmation blocks after a transaction [20,21].

Some work has been done to introduce sharding for cryptocurrencies [22–24]. If the validation of transactions could be securely distributed and every node only had to process a part of all transactions, the transaction rate could scale linearly with the number of nodes. One especially interesting approach has been published as our submission of the conference version of this work, called Plasma [25]. Plasma has the property that the members of a shard are the same people that care about its contents, similar to payment channels. Indeed, one could also interpret payment channels as interest-based shards of a blockchain. Plasma also introduces trees of blockchains, splitting interest groups into smaller subgroups. The same hierarchical structure has been introduced to payment channels with this work!

### 5.1. Payment networks

Solutions to find routes through a payment network in a scalable and decentralized way have been proposed, based on central hubs [26], rotating global beacons [27], personal beacons, where overlaps between sender and receiver provide paths [28], or combinations of multiple schemes [29].

**Figure 14.**
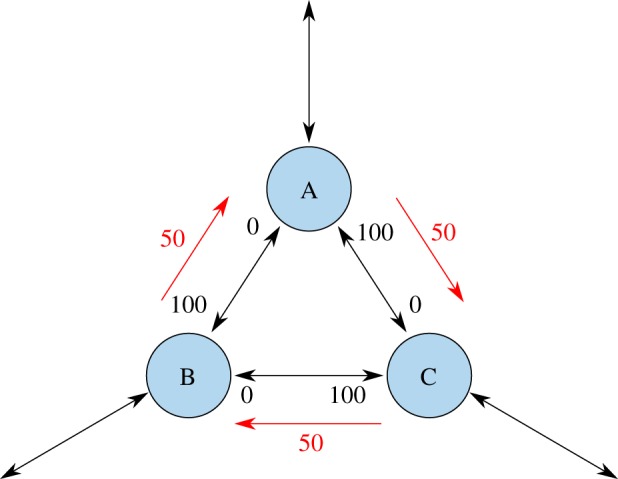
Rebalancing a cycle of channels, which have become one sided. The channels between A, B and C have been heavily used in one direction, e.g. external transactions being routed counterclockwise. As a result, one direction of each channel cannot be used any more due to insufficient funds. An atomic cyclic transfer, shown by the red arrows, can turn the three channels usable again. The transaction does not change the total stake of any involved party.

A known way to rebalance channels in a payment network are cyclic transactions, shown in figure 14. The idea has originated in private communication between the developers of the Lightning Network.^6^

While cyclic rebalancing allows us to reset channels which have run out of funds, it has limitations. If the amount of funds running through a specific edge has been estimated wrong at funding time, or changes over time, rebalancing might become necessary frequently. This slows down transactions which have to wait for the rebalancing to finish. Our solution with channel factories allows moving the locked-in funds to a different channel to solve the problem for a longer time.

## 6. Differences to the conference version

**Figure 15.**
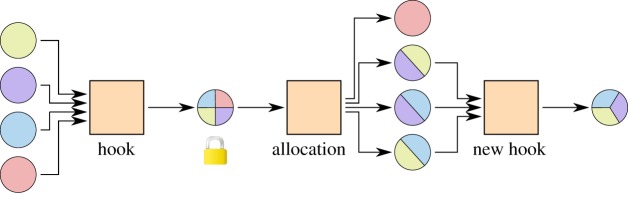
A set-up to merge subchannels into a larger channel again. It is insecure because two parties can close their subchannels with a different transaction without agreement from the third party and make the new hook impossible to execute.

This work was presented in an earlier version at the 19th International Symposium on Stabilization, Safety, and Security of Distributed Systems [30]. Apart from small extensions, a section was removed as a flaw was discovered. The section contained an idea to re-merge subchannels into a larger channel in further unpublished transactions similar to figure 15. However, this is insecure, as any owners of one of the merged subchannels can close their subchannel with a different transaction than the new hook at any time. This means the new hook does not provide the secure lock-in feature required to base payment channels of it.

From this, we learn that any transaction that is a descendent of an unconfirmed transaction can only be trusted by people that are also required to sign everything that could replace the unconfirmed transaction. In other words, to trust a transaction one has to make sure that every unconfirmed ancestor transaction requires one's signature to be replaced. This also means channels can only have less participants further down the transaction graph.

## 7. Conclusion

We introduced a new layer of channel factories, sitting between the blockchain and the network of micropayment channels. Within a group of nodes, channel factories allow for more flexibility, creating many micropayment channels without additional blockchain usage, and easy movement of locked-in funds to other subchannels of the same factory using only off-blockchain collaboration. By creating many of those channel factories with some member overlap, a network of micropayment channels can be created with a lower use of blockchain space compared to existing systems.

The larger a group, the more space is saved, as the additional channels amortize the blockchain transactions. Three-party channel factories save 50% of the blockchain space. In a setting of 20 users with 100 channels between them, 90% reduction is achieved. In a Bitcoin system with signature aggregation those numbers improve even more to 62% and 96%, respectively.

With a larger number of nodes in a channel factory, there is an increased risk of someone closing the channel factory, creating blockchain transaction costs for everyone involved; however, there is no gain for the acting party, meaning that any entity that is trusted to act selfishly will be a good channel factory member. Nevertheless, this risk limits the usefulness of large groups.

## Footnotes

1 This is just a regular transaction without any special script. Its inclusion in the blockchain just ‘kicks off’ the timers on the subsequent transactions, as they are relative to the previous transaction.

2 The original publication preceded the introduction of relative timelocks and as a result had to use a different tree.

3 A cold wallet is a wallet stored on a device that is most of the time disconnected from the Internet and acts as a secure long-term storage for funds.

4 See Schnorr signatures at https://bitcoincore.org/en/2016/06/24/segwit-next-steps/.

5 ed25519 is not the only possible implementation of Schnorr signatures. If you prefer the implementation based on curve secp256k1, just calculate with a 33 byte public key instead of 32.

6 It is mentioned in https://lists.linuxfoundation.org/pipermail/lightning-dev/2015-September/000188.html.

## Data accessibility

This article has no additional data.

## Appendix A. Scripts

This appendix lists the different Bitcoin scripts to implement the proposed system. For every output, there is one script that describes the conditions to claim the output and another one that fulfils those conditions and is provided in the input. These scripts depend on a deployed fix for malleability, e.g. Segregated Witness. Note that an implementation might move the output script into the input and use a hash commitment to ensure its integrity and authenticity as usually done in P2SH Bitcoin transactions.

**A.1. Two-party channel with timelocks**

These scripts implement the transactions in figure 5.

The funding and kickoff transaction and all transactions in the invalidation tree apart from the leaves have simple multisig outputs, as shown in Script 1.

**Table:**

**Script 1.** Simple two-party multisignature output.
2 <pubkey A> <pubkey B> 2 OP_CHECKMULTISIG

All these outputs can be spent with Script 2 in the input.

**Table:**

**Script 2.** Input script to spend a simple two-party multisignature output.
0 <sig A> <sig B>

The transactions in the invalidation tree have an additional timelock on the input. This is achieved by setting the nSequence field of the input to the required time according to BIP68 [8]. These are then automatically enforced by the miners. The locktime is smaller each time a transaction is replaced and thus a new branch in the tree created.

The leaves of the invalidation tree split the funds into two simple P2PKH outputs.

**A.2. Multi-party channel with timelocks**

These scripts implement the timelock-based multi-party channel in figure 7. Assume p parties. The multisig outputs in the funding, kickoff and invalidation tree transactions before the split into the subchannels are regular p-party multisignature outputs, Script 3.

**Table:**

**Script 3.** p-party multisignature output
p <pubkey 1> <pubkey 2> … <pubkey p> p OP_CHECKMULTISIG

They are all spent with Script 4 in the inputs.

**Table:**

**Script 4.** Input script to spend a p-party multisignature output
0 <sig 1> <sig 2> … <sig p>

The transactions of the invalidation tree have additional timelocks, again implemented by setting the nSequence field in the inputs according to BIP68 [8].

The leaves of the tree have any number of outputs, each creating a two-party subchannel with Script 1.
